# Efficacy and Safety of Sotatercept in Pulmonary Artery Hypertension :A Meta-analysis of Randomized Control Trials

**DOI:** 10.51894/001c.163959

**Published:** 2026-08-01

**Authors:** Romman Fatima FNU, Muhammad Ayyan

**Affiliations:** 1 Detroit Medical Center - Sinai Grace Hospital

## 04

### BACKGROUND

Pulmonary arterial hypertension (PAH) is a progressive disease characterized by pulmonary vascular remodeling, increased pulmonary vascular resistance, and right ventricular failure, resulting in significant morbidity and mortality. Sotatercept, a novel activin/TGF-β pathway modulator, has emerged as a promising therapy targeting vascular remodeling. Prior systematic reviews evaluating its efficacy predate the pivotal HYPERION trial. This systematic review and meta-analysis aimed to evaluate the efficacy and safety of sotatercept in adults with PAH.

### METHODS

Following PRISMA guidelines, we conducted a systematic review and meta-analysis of randomized controlled trials evaluating sotatercept in adults with PAH. MEDLINE, Embase, CENTRAL, and ClinicalTrials.gov were searched through February 2026. Eligible studies compared sotatercept plus background therapy with placebo or standard care. Primary outcomes included 6-minute walk distance (6MWD), pulmonary vascular resistance (PVR), and time to clinical worsening. Secondary outcomes included NT-proBNP, WHO functional class, mortality, and safety. A random-effects model was used for pooled analyses.

### RESULTS

Sotatercept significantly reduced clinical worsening (RR 0.26, 95% CI 0.17–0.38) and improved 6MWD (MD 31.0 m, 95% CI 15.09–46.94) and WHO functional class (RR 2.04, 95% CI 1.53–2.70). Significant improvements were observed in PVR (MD −201.5 dyn·s·cm⁻⁵), mean pulmonary artery pressure (MD −14.92 mmHg), right atrial pressure (MD −2.58 mmHg), and NT-proBNP (MD −271.49 pg/mL). Mortality was similar between groups. Sotatercept reduced cardiac events and serious adverse events but increased hemoglobin levels, telangiectasia, bleeding, and hypertension.

### CONCLUSIONS

Sotatercept improves exercise capacity, pulmonary hemodynamics, and functional status in PAH with a manageable safety profile, supporting its role as an effective add-on therapy.

